# Definitive Upfront Stereotactic Ablative Radiotherapy Combined with Image-Guided, Intensity Modulated Radiotherapy (IG-IMRT) or IG-IMRT Alone for Locally Advanced Non-Small Cell Lung Cancer

**DOI:** 10.1371/journal.pone.0162453

**Published:** 2016-09-09

**Authors:** Alexander Chi, Sijin Wen, Manish Monga, Mohammed Almubarak, Xiaoqing He, Yon Rojanasakul, William Tse, Scot C. Remick

**Affiliations:** 1 Mary Babb Randolph Cancer Center of West Virginia University, Morgantown, WV, United States of America; 2 Department of Biostatistics, School of Public Health, West Virginia University, Morgantown, WV, United States of America; 3 Oncology, Bristol Meyers Squibb, Princeton, NJ, United States of America; 4 Division of Hematology & Oncology, Mary Babb Randolph Cancer Center of West Virginia University, Morgantown, WV, United States of America; 5 Department of Pharmaceutical Sciences, West Virginia University, Morgantown, WV, United States of America; 6 Department of Hematology, James Graham Brown Cancer Center of the University of Louisville, KY, United States of America; North Shore Long Island Jewish Health System, UNITED STATES

## Abstract

**Background:**

Image-guided (IG) intensity-modulated radiotherapy (IMRT) enables maximal tumor margin reduction for the sparing of organs at risk (OARs) when used to treat locally advanced non-small cell lung cancer (NSCLC) with definitive chemo-radiation. It also allows for the incorporation of stereotactic ablative radiotherapy (SABR) into the treatment regimen. Here, we describe our initial experience in combining definitive upfront SABR to the primary lesion with chemo-radiation delivered with conventionally fractionated IG-IMRT to the remaining regional disease; along with clinical outcome following chemo-radiation with conventionally fractionated IG-IMRT alone in the treatment of locally advanced NSCLC.

**Methods:**

The clinical outcome of 29 patients with locally advanced NSCLC who underwent conventionally fractionated IG-IMRT, or definitive upfront SABR followed by IG-IMRT combined with chemotherapy (induction, concurrent, or both) was retrospectively reviewed.

**Results:**

After a median follow up of 23.7 months, the median overall survival (OS) and progression-free survival (PFS) were 19.8 and 11.3 months, respectively. The 2 year local, regional, and distant control was 60%, 62%, and 38%, respectively. No local failure was observed in 3 patients following SABR + IG-IMRT while 6/26 patients failed locally following IG-IMRT alone. SABR + IG-IMRT was well tolerated. No ≥ grade 3 radiation-related toxicity was observed.

**Conclusion:**

Definitive upfront SABR followed by IG-IMRT in selected patients with locally advanced NSCLC warrants further investigation in future clinical trials, while chemo-radiation with IG-IMRT alone was well tolerated.

## Introduction

In recent years, advances in technology such as 4DCT and intensity modulated radiotherapy (IMRT) led to lower incidence of radiation-related toxicities and better short-term survival in the treatment of locally advanced non-small cell lung cancer (NSCLC) with chemo-radiation when compared to 3D techniques [1, 2]. Treatment accuracy and thoracic OAR sparing can be further improved with daily image guidance due to more accurate tumor localization and the safe PTV margin reduction it allows [3]. At the current time, image guided (IG)-IMRT may represent one of the best radiotherapy delivery approaches in the treatment of locally advanced lung cancer. With its advantages in OAR sparing, various strategies for radiation dose escalation in the thorax become clinically feasible. As previously shown, dose escalation may increase the tumor control probability in patients with locally advanced NSCLC, possibly leading to improved survival [4, 5, 6]. Thus, effective dose escalation with IG-IMRT may represent an important strategy to improve the clinical outcome in these patients. Given the negative results obtained from RTOG 0617, a phase III randomized study assessing the benefit of moderate dose escalation (conventionally fractionated) with 3D conformal radiotherapy (3D-CRT) or IMRT in the patients receiving chemo-radiation for unresectable stage III NSCLC, similar strategies of dose escalation with IG-IMRT has not been actively pursued. In RTOG 0617, patients were randomized to chemo-radiation to 60 Gy vs. 74 Gy, and with or without Cetuximab [7]. While no survival benefit was obtained with the addition of cetuximab to the treatment regimen in general, dose escalation resulted in inferior median survival (20.3 *vs*. 28.7 months, *p* = 0.004) and no improvement in local control at 2 years (61.4% *vs*. 69.3%, *p* = 0.13). The causes of poorer outcome in the 74-Gy arms remain to be discerned. In theory, local control may be significantly decreased by delayed tumor cell repopulation associated with prolonged overall treatment time, which may be one reason for the lack of clinical benefit observed with moderate, conventionally-fractionated dose escalation [8]. This problem may be solved by adopting alternative dose escalation strategies, such as stereotactic ablative radiotherapy (SABR), to deliver a high dose to the tumor over a shorter overall treatment time course. This is well evidenced by the clinical success of stereotactic ablative radiotherapy (SABR) in the treatment of early stage NSCLC [9]. One unique strategy is to increase the tumor BED at the primary site with definitive SABR, which is followed by conventionally fractionated chemo-radiation to the remaining regional disease ± separate primary lesions in the same or other lung lobes with IG-IMRT in certain patients with non-bulky regional nodal disease. In this study, we describe our initial experience with this treatment approach along with our clinical experience with chemo-radiation delivered with conventionally fractionated IG-IMRT.

## Materials and Methods

### Patient Selection

Twenty nine consecutive patients with stage II-IV NSCLC treated with IG-IMRT, including 3 patients treated with definitive upfront SABR to the primary site followed by IG-IMRT to the remaining disease, in the Department of Radiation Oncology of West Virginia University (WVU) between October, 2012 and May, 2015 were included. This study was approved by the WVU Institutional Review Board under WVU research corporation office of research integrity & compliance, and informed consent was not required due to its retrospective nature. Although written consent was not signed by any patient, patient information was anonymized and de-identified prior to any analysis. All patients’ diagnoses were pathologically confirmed. All patients were staged with fluoro-deoxyglucose positron emission tomography-computed tomography (FDG PET/CT), and IV contrasted CT or MRI of the brain. The tumor, node, and metastases staging system of the 7^th^ edition of the American Joint Committee for Cancer Staging System was used for staging (AJCC 2010).

### Treatment Planning

All patients treated with IG-IMRT alone were simulated supine while immobilized in alpha cradles (Vac-Lok, CIVCO Medical Solutions, Coralville, IA) with the use of T-bars, wing boards, and support for the head, shoulders, arms, and lower extremities. For patients who were treated with SABR + IG-IMRT, they were immobilized with abdominal compression in a dedicated SBRT immobilization device (CIVCO Medical Solutions, Coralville, IA) for both portions of their radiotherapy with the use of T-bars, wing boards, and supports for the head, shoulders, arms, and lower extremities. Patients were simulated with 4D CT or 4D FDG PET/CT. Planning CTs were acquired with 3-mm slices. The gross tumor volume (GTV) was delineated on the non-contrasted, free-breathing treatment planning CT. The internal target volume (ITV) was contoured to include the tumor from all 10 phases of the 4D CT or 4D FDG PET/CT. Free breathing PET/CT was fused to the planning CT for ITV/ CTV delineation if 4D PET/CT was not done. For IG-IMRT, a 5–10 mm expansion was used to create the CTV. The planning target volume (PTV) was the CTV plus a 3–5 mm expansion. For SABR, ITV was directly expanded by 3–5 mm to create the PTV. The lungs, esophagus, spinal cord, and the heart were contoured for each patient. Major vessels and airway were delineated if necessary. Target volume delineation and treatment planning were both performed in the Eclipse treatment planning system (Varian Medical Systems, Palo Alto, CA).

### Treatment Delivery

**Fig 1:**
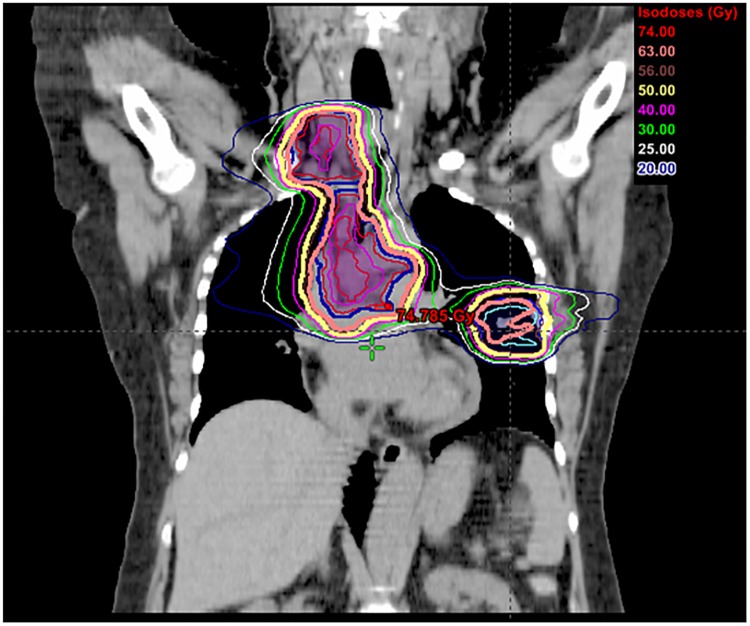
Illustration of a composite treatment plan for a patient with oligo-metastatic poorly differentiated squamous cell carcinoma (cT1a, N3, M1b). She received upfront carboplatin/gemcitabine with excellent extra-thoracic response. Subsequently, SABR to her primary disease + IG-IMRT to her regional disease as the only remaining disease following chemotherapy were administered. She remains disease free 2 years after the completion of all treatments.

Conventionally fractionated IG-IMRT treatments were delivered with either volumetric modulated arc therapy (VMAT) using Rapid Arc, or regular multi-field IMRT with dynamic multi-leaf collimator (MLC). 60–70 Gy (median: 63 Gy) was delivered in daily fractions of 1.8–2 Gy with 6–10 MV photons. The majority of patients received concurrent chemotherapy with conventionally fractionated IG-IMRT. OAR dose constraints used for IG-IMRT were the following: total lung, V_5_ < 65%, V_20_ < 35%, mean lung dose (MLD) < 19 Gy; spinal cord, maximum dose < 45 Gy; heart, V_30_ < 50%, mean dose ≤ 35 Gy; esophagus, V_55_ < 50%, mean dose ≤ 34 Gy, maximum dose < 105% of the prescription dose. For the 3 patients who received SABR & IG-IMRT (Fig 1), SABR was delivered prior to IG-IMRT with 6 MV photons. The dose was 40–50 Gy delivered in 4 daily fractions. No systemic therapy was given during SABR delivery. For these patients, the dose for conventionally fractionated IG-IMRT was 63 Gy delivered in 35 daily fractions. For SABR, the OAR dose was kept to as low as possible to keep the composite maximum dose to the spinal cord to ≤ 50 Gy, the esophagus and the heart to ≤ 70 Gy; and the composite MLD to < 20–21 Gy. All treatments were delivered under daily image guidance with cone beam CT.

### Follow Up

The patients were followed after 4 weeks, then every 3 months from the completion of all radiotherapy with a history and physical examination, FDG PET/CT or CT of the chest every 3 months, and periodical basic laboratory studies, including the comprehensive metabolic panel, complete blood count, and liver function tests for 2 years then every 3–6 months thereafter for 3 years. Treatment-related toxicity was assessed during each follow up visit with the National Cancer Institute Common Terminology Criteria for Adverse Events (NCI-CTCAE) v4.0. Additional evaluation, including biopsies of suspected recurrence/metastasis, was done if indicated. Pulmonary function tests are recommended to all patients 3 months after completion of their radiotherapy.

### Statistical Analysis

Descriptive statistical analysis was used to summarize the patients' characteristics, including contingency tables with counts and percentages for categorical variables; mean (± s.d.) or median (range) for continuous variables. Boxplot was used to summarize the heart’s mean dose (MD), and the heart volume receiving 5 Gy (V_5_), 30 Gy (V_30_), and 40 Gy (V_40_) between patient subgroups. Kaplan-Meier method was used to examine overall survival and progression-free survival functions including local, reginal and distant relapses. Log-rank test was used to assess the difference of time-to-event data between patient subgroups including stages, mutation (yes/no), dose (high/low), and other baseline characteristic. A *p*-value < 0.05 was considered statistically significant. Statistical analyses were carried out using SAS 9.1 (SAS Institute, Cary, NC) and S-Plus, version 7.0 (Insightful Corp., Seattle, WA) software.

## Results

### Patient Characteristics

Patients’ clinical and treatment characteristics are listed in Table 1. 29 patients with primary stage IIA-IIIB & oligo-metastatic stage IV, or recurrent NSCLC were included in this study. Among them, 93.1% were stage III (primary, or recurrent). The 4 patients with recurrent disease were initially treated with surgery ± adjuvant chemotherapy for stage I-III NSCLC. 26 patients received conventionally fractionated IG-IMRT alone, while 3 patients were treated with SABR to the primary site combined with IG-IMRT to the remaining sites of disease. The majority of patients received concurrent platinum-based chemotherapy during IG-IMRT (25 patients) or platinum-based chemotherapy prior to any radiotherapy (4 patients). Among the patients who received concurrent chemotherapy, 12 patients also received induction chemotherapy. Two of the patients who were treated with SABR + IG-IMRT underwent chemotherapy prior to any radiotherapy; while one received concurrent chemotherapy with IG-IMRT after SABR delivery was completed.

**Table 1:** Patient Demographics and Clinical Characteristics (n = 29 patients).

Characteristics	# Patients (%)
**Median Age (range)**	66 (51–81)
**Sex**	
Male	14 (48.3%)
Female	15 (51.7%)
**ECOG performance score**	
0–1	25 (86.2%)
2	4 (13.8%)
**Current/ former cigarette smoking**	28 (96.6%)
**Tumor stage (primary disease)**	
IIA	1 (3.4%)
IIIA	11 (37.9%)
IIIB	12 (41.4%)
IV(oligo-metastatic)	1 (3.4%)
Recurrent*	4 (13.8%)
**Tumor histology**	
Adenocarcinoma	15 (51.7%)
Squamous cell carcinoma	13 (44.8%)
Large cell carcinoma	1 (3.4%)
**Median Radiation dose (range)**	
Image-guided IMRT	63 (60–70) Gy
SABR	50 (40–50) Gy
**Treatment technique**	
Image-guided IMRT	
* VMAT*	18 (62.1%)
* Multi-field IMRT*	8 (27.6%)
SABR (VMAT) + VMAT (#1) or IMRT (#2)	3 (10.3%)
**Median treatment duration, minutes (range)**	
Image-guided IMRT	4.21 (1.81–12.67)
* VMAT*	3.72 (2.14–9.96)
* Multi-field IMRT*	5.05 (3.12–12.67)
SABR	17.55 (6.0–19.67)
**Chemotherapy**	
Upfront (Sequential or Induction)	16 (55.2%)
Concurrent	25 (86.2%)
**Median follow Up, months (range)**	23.7 (5.3–32.0)

*2 recurrent IIIA, 2 recurrent IIIB

### Clinical Outcome

**Fig 2:**
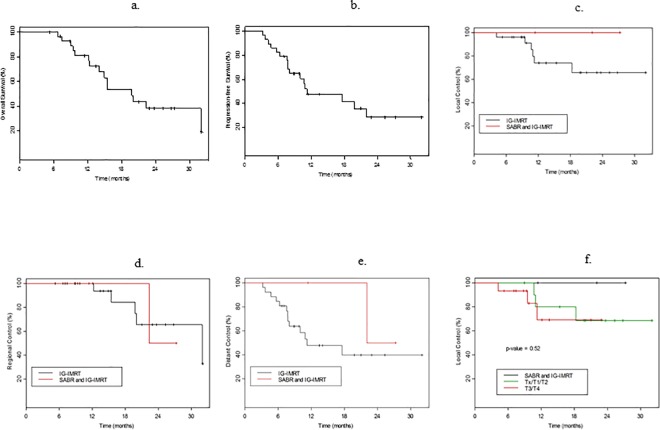
The a) overall survival (OS), and b) progression-free survival (PFS) for all patients. The c) local, d) regional, and e) distant control for patients who were treated with SABR ± IG-IMRT. f) Local control following SABR + IG-IMRT vs. that in patients with T_x_, T1/2 or T3/4 disease who received IG-IMRT.

For all 29 patients, the median overall survival (OS) and progression-free survival (PFS) after a median follow up of 23.7 months were 19.8 months and 11.3 months, respectively (Fig 2A and 2B). As shown, the 2-year OS and PFS were 38% and 29%. The majority of deaths were due to distant progression (12/15 patients). Overall, the local, regional, and distant control rates at 2 years were 60%, 62%, and 38%, respectively. No local failure was observed in the 3 patients who received definitive upfront SABR combined with IG-IMRT. Six local failures were observed in patients who were treated with the conventionally fractionated IG-IMRT alone combined with chemotherapy (concurrent, induction, or both). One of the three patients who received SABR + IG-IMRT experienced regional and distant failures. Five patients experienced regional failures, and 13 patients experienced distant failures following IG-IMRT alone. The local, regional, and distant control for patients who received SABR + IG-IMRT and those for patients who received IG-IMRT are shown in Fig 2C–2E.

### Treatment-related Toxicity

Definitive upfront SABR + IG-IMRT was well tolerated without any acute or late ≥ grade 3 toxicity observed in all three patients. In the 26 patients who were treated with conventionally fractionated IG-IMRT, no radiation-related pneumonitis was observed while the incidence of grade 1–2 acute esophagitis was 53.8%. Grade 1–2 acute erythema in the treated areas was also observed in 2 patients. No late toxicity was observed. The dosimetric factors for all patients are shown in Table 2.

**Table 2:** Dosimetric parameters for patients who received SABR + IG-IMRT (n = 29 patients).

	IG-IMRT alone	SABR + IG-IMRT		
		IG-IMRT	SABR	Composite
PTV dose coverage^*^				
V_100_	95% (85%–100%)	96% (91%–100%)	96% (95%–99%)	
V_95_	100% (94%–100%)	100% (100%–100%)	100% (100%–100%)	
D_95_	100% (85%–103%)	100% (95%–102%)	101% (100%–103%)	
D_max_	114% (108%–128%)	110% (108%–119%)	125% (123%–125%)	
D_min_	82% (56%–97%)	94% (90%–99%)	93% (91%–95%)	
D_mean_	105% (102%–112%)	105% (104%–105%)	111% (110%–111%)	
OAR doses^*^				
*Total lung dose*				
MLD	18.10 (7.42–20.73) Gy	11.26 (10.66–15.96) Gy	2.96 (1.45–5.02) Gy	16.28 (13.62–17.41) Gy
V_5_	64% (52%–76%)	55% (42%–62%)	10% (7%–19%)	63% (54%–64%)
V_10_	49% (17%–56%)	41% (34%–47%)	8% (3%–15%)	49% (43%–56%)
V_20_	31% (4%–36%)	21% (19%–29%)	4% (1%–9%)	31% (24%–37%)
*Spinal cord*				
D_max_	43.17 (28.45–44.74) Gy	36.51 (36.27–38.00) Gy	7.84 (3.23–8.86) Gy	36.88 (36.42–39.32) Gy
*Esophagus*				
D_max_	70.02 (53.59–76.39) Gy	60.47 (52.86–65.87) Gy	7.49 (4.13–10.15) Gy	63.76 (53.00–67.99) Gy
D_mean_	28.00 (9.66–41.45) Gy	14.85 (12.97–22.01) Gy	1.01 (0.58–1.16) Gy	15.43 (13.98–23.17) Gy
V_55_	27% (0%–55%)	1% (0%–16%)	0% (0%–0%)	1% (0%–17%)
*Heart*				
D_max_	—	63.66 (54.61–65.78) Gy	11.44 (0.29–35.66) Gy	65.66 (55.18–65.83) Gy
D_mean_	14.56 (2.61–37.00) Gy	3.38 (2.92–10.44) Gy	1.65 (0.04–5.42) Gy	8.34 (5.03–10.49) Gy
V_5_	61% (9%–100%)	15% (9%–52%)	7% (0%–41%)	52% (29%–67%)
V_30_	17% (0%–55%)	2% (1%–9%)	0% (0%–0.2%)	2% (1%–9%)
V_40_	10% (0%–46%)	1% (0.5%–3.49%)	0% (0%–0%)	1% (1%–4%)
*Major blood vessels*				
D_max_	—	69.05 (60.97–70.89) Gy	6.68 (5.05–11.47) Gy	74.30 (61.25–75.01) Gy
*Major airway*				
D_max_	—	66.30 (58.22–71.84) Gy	5.18 (0.47–6.23) Gy	69.27 (58.61–75.55) Gy

*median (range); D_95_, D_max_, D_min_, and D_mean_ for the PTV are presented as a percentage of the prescription dose.

## Discussion

In this study, the median survival was 19.8 months for all 29 patients with locally advanced NSCLC. It appears to be similar to that observed in the MD Anderson study (1.8 years) on patients with stage III-IV NSCLC treated to a median dose of 66 Gy in 33 daily fractions with IMRT ± chemotherapy [2]. The local control was 60% at 2 years for all patients, which is similar to that observed in another study on IMRT ±chemotherapy to an average dose of 69.5 Gy for stage I-III NSCLC, the 2 year local control and OS in 39 patients with stage IIIA/B NSCLC were 58% and 58%, respectively [10]. Despite similar local control, worse OS at 2 years was observed in this study. However, no conclusion can be made regarding to the efficacy of IG-IMRT due to this study’s small sample size. Nevertheless, the median survival appears to be better than that observed in most phase III chemo-radiation trials for locally advanced NSCLC, which is usually less than 18 months [3]. As shown in RTOG 9410, a phase III randomized study assessing the benefit for concurrent vs. sequential chemo-radiation with 3D techniques; the best median survival achieved was 17 months following concurrent chemo-radiation [11]. Our overall regional and distant control rates were 62% and 38% at 2 years. The high incidence of regional and distant failures may be associated with the presence of bulky regional nodal disease and the inclusion of patients with relatively more advanced disease. High incidence of distant metastases following definitive chemo-radiation is fairly common in patients with locally advanced NSCLC, which will need to be better addressed as lung cancer therapeutics evolves in the future.

Patients who received definitive upfront SABR + IG-IMRT did not experience any severe toxicity. No local failure following SABR was observed, while 6 of 26 patients who received IG-IMRT alone failed locally. This suggests a local control benefit of delivering a high BED to the primary tumor over a short period of time. As shown in the treatment of early stage NSCLC with SABR, tumor BED of ≥ 100 Gy_10_ may lead to local control of > 90%, and increase the OS in patients who are surgically operable [12]. When feasible, definitive upfront SABR + IG-IMRT may also be more effective than other regimens of moderate hypo-fractionation in achieving a high rate of local control [5, 13–15]. This may potentially augment the efficacy of conventional IG-IMRT in locally advanced NSCLC. One of the 3 patients experienced a regional failure and brain metastases simultaneously 15 months after completion of sequential chemo-radiation. She subsequently died from disease progression. Her regional disease was initially treated to 63 Gy in 35 daily fractions, which may be suboptimal for the control of her regional disease. Our exploratory findings suggest that definitive upfront SABR to the primary tumor followed by conventionally fractionated IG-IMRT to the remaining disease, enabled by image guidance and intensity modulation, may be feasible in selected patients with locally advanced NSCLC undergoing chemo-radiation. Its efficacy opt to be further investigated in future clinical trials. Although the interplay between IMRT and respiratory motion has been a concern, this can be mitigated through various motion management techniques, such as the motion encompassing approach with 4D CT as previously discussed [16].

Factors such as large PTV and KRAS mutations in adenocarcinomas (all on codon 12) were found to be associated worse OS (p < 0.05). PTV directly correlates with the tumor size, which has been known to be associated with local control and survival [17, 18]. The negative prognostic value of certain KRAS mutations in NSCLC, and their association with radio-resistance have long been known [19–22]. The likely mechanism is the constitutive activation of its downstream pathways, leading to increased survival of tumor cells following irradiation [21, 22]. Furthermore, the MD (mean dose) of the heart, and volumes of the heart receiving 5 Gy, 30 Gy, and 40 Gy (V_5_, V_30_, and V_40_) for patients who were alive appeared to be lower than that for patients who already died from disease progression. While no conclusion can be made due to the small size of the current study, the implications of these findings warrant further investigation in larger studies.

As an exploratory study on the feasibility of definitive upfront SABR to be followed by chemo-radiation delivered with IG-IMRT in the treatment of locally advanced NSCLC, its major limitation is the small sample size. However, this study not only explores the potential feasibility of a unique approach to treat locally advanced NSCLC, but also reports our institutional experience with IG-IMRT in the treatment of locally advanced NSCLC without making any firm conclusions. Validation studies with larger sample size will be conducted in the future to further define the role of definitive SABR + IG-IMRT in the treatment of locally advanced NSCLC.

## Conclusion

Our study demonstrates that definitive upfront SABR + IG-IMRT in the setting of chemo-radiation for locally advanced NSCLC may be feasible and warrants further validation in future clinical trials while conventionally fractionated IG-IMRT was associated with excellent tolerability.
